# Resveratrol Protects against 2-Bromopropane-Induced Apoptosis and Disruption of Embryonic Development in Blastocysts

**DOI:** 10.3390/ijms12084991

**Published:** 2011-08-05

**Authors:** Wen-Hsiung Chan

**Affiliations:** Department of Bioscience Technology and Center for Nanotechnology, Chung Yuan Christian University, Chung Li 32023, Taiwan; E-Mail: whchan@cycu.edu.tw; Tel.: +886-3-2653515; Fax: +886-3-2653599

**Keywords:** resveratrol, 2-Bromopropane, blastocyst, apoptosis, development

## Abstract

2-Bromopropane (2-BP) is used as an alternative to ozone-depleting cleaning solvents. Previously, we reported that 2-BP has cytotoxic effects on mouse blastocysts and is associated with defects in subsequent development. In the present work, we show that 2-BP induces apoptosis in the inner cell mass of mouse blastocysts, and inhibits cell proliferation. Both effects are suppressed by resveratrol, a grape-derived phytoalexin with known antioxidant and anti-inflammatory properties. 2-BP-treated blastocysts displayed lower levels of implantation (compared to controls) when plated on culture dishes *in vitro,* and a reduced ability to proceed to later stages of embryonic development. Pretreatment with resveratrol prevented 2-BP-induced disruption of embryonic development, both *in vitro* and *in vivo*. Further investigation of these processes revealed that 2-BP directly promotes ROS generation, loss of mitochondrial membrane potential (MMP), and activation of caspase-3, whereas resveratrol effectively blocks 2-BP-induced ROS production and the accompanying apoptotic biochemical changes. Our results collectively imply that 2-BP triggers the mitochondrion-dependent apoptotic pathway via ROS generation, and the antioxidant activity of resveratrol prevents 2-BP-induced toxicity.

## 1. Introduction

2-Bromopropane (2-BP), a cleaning agent, is used as an alternative to ozone-depleting solvents. In 1995, 2-BP caused a series of reproductive and hematopoietic disorders in both female and male workers exposed to the material. The solvent was commonly used in an electronics factory located in South Korea [1,2]. Moreover, earlier reports found a high incidence of oligozoospermia in male workers after long-term exposure to 2-BP [1–3]. Several animal studies have further confirmed the potential of 2-BP to injure the reproductive, hematopoietic, central nervous, and immune systems [4–11]. In cytotoxicity experiments, mouse embryos treated with 2-BP displayed micronuclear formation and a decrease in embryo cell number [12]. Moreover, 2-BP was recently identified as a potent DNA damaging agent [5,8]. These results collectively suggest that 2-BP induces various toxicities via DNA damage. A reproductive toxicity investigation further demonstrated that exposure to 2-BP induced testicular or ovarian dysfunction, causing injury to early types of spermatogenic cells or primordial follicles and oocytes of rats [4,6]. In experiments investigating the effects of 2-BP on pre- and postnatal development, exposure of pregnant or lactating female rats to 2-BP resulted in delivery rate decrease, peri- and postnatal death increase, loss of body weight development, and higher incidence of reproductive organ dysfunction [13]. It is true that, to date, no clinical or epidemiological study, or case report, has demonstrated a direct relationship between exposure of pregnant workers to 2-BP and reproductive problems. Specifically, there is no evidence to suggest that the solvent negatively affects embryonic development or infant growth. However, it is very important to explore the health risks associated with exposure of female workers, especially those who are pregnant, to 2-BP. Importantly, the solvent is very volatile and can permeate human skin. The major exposure route is via inhalation in the workplace or factory [1,2]. Moreover, a recent study by our group showed that 2-BP induces cellular apoptosis in both the ICM and TE of mouse blastocysts, leading to a decrease in implantation, a reduction in embryonic development, and a loss of embryo viability. Furthermore, our recent study results indicate that 2-BP induces developmental injury via induction of cell apoptosis processes in oocyte maturation and early-stage embryos [14]. These findings clearly suggest that short-term exposure to 2-BP is a risk factor for normal mouse embryonic development, and may inhibit oocyte maturation in infertile subjects.

Resveratrol, a member of the phytoalexin family, found in grapes and other dietary plants, inhibits tumor initiation and progression [15–17]. Resveratrol exerts a wide range of pharmacological effects, including prevention of heart disorders, blocking of lipoprotein oxidation, and inhibition of platelet aggregation [18–20]. The anti-tumor properties of resveratrol have been attributed to an antioxidant activity and an ability to inhibit activation of cyclooxygenases [15,21,22], and may also be associated with a capacity to cause cell cycle arrest and apoptosis [23–27]. In earlier experiments by our group, pretreatment with resveratrol prevented ethanol-induced disruption of embryonic development both *in vitro* and *in vivo* [28]. Our results indicated that resveratrol reduces ethanol-induced injury (including apoptosis), inhibition of cell proliferation, and retardation of embryonic development, because of the antioxidant properties of the material [28]. Although several biological functions of resveratrol have thus been established, the effects of the material on 2-BP-triggered injury, and the underlying mechanisms thereof, are currently unknown.

To explore whether resveratrol prevents 2-BP-induced injury to embryos, we examined the effect of the compound on apoptosis, proliferation, and blastocyst development. Pretreatment with resveratrol effectively suppressed 2-BP-induced injury, including cell apoptosis, inhibition of cell proliferation, and retardation of embryonic development, both *in vitro* and *in vivo*. Further studies were conducted to elucidate the mechanisms underlying these effects.

## 2. Results and Discussion

**Figure 1.**
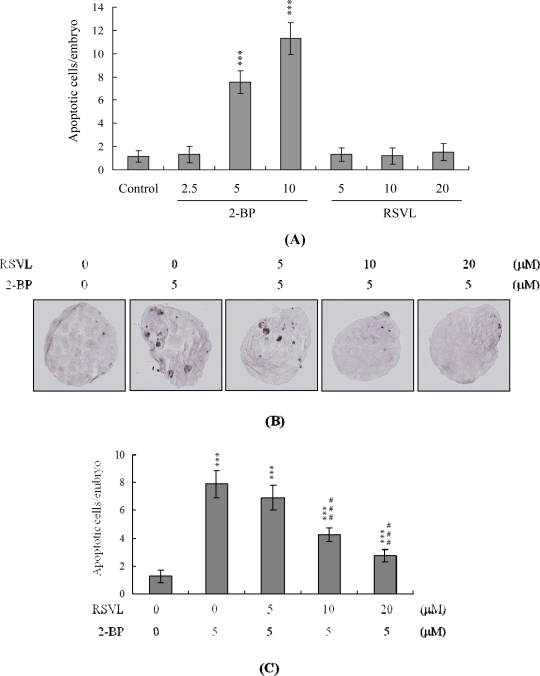
Effect of resveratrol on 2-Bromopropane (2-BP)-induced apoptosis in blastocysts. (**A**) Mouse blastocysts were incubated with resveratrol (RSVL; 5–20 μM) or 2-BP (2.5–10 μM) for 24 h. TUNEL-stained cells in blastocysts were visualized under a light microscope. The mean number of apoptotic (TUNEL-positive) cells per blastocyst was calculated. (**B** and **C**) Mouse blastocysts were preincubated with RSVL (5–20 μM) for 1 h, followed by treatment with or without 5 μM 2-BP for another 24 h. Cells were visualized using light microscopy. TUNEL-positive cells are depicted in black (B). The mean number of TUNEL-positive cells per blastocyst was calculated (C). *** *P* < 0.001 *vs.* the control group; ^###^ *P* < 0.001 *vs.* the 5 μM 2-BP-treated only group.

**Figure 2.**
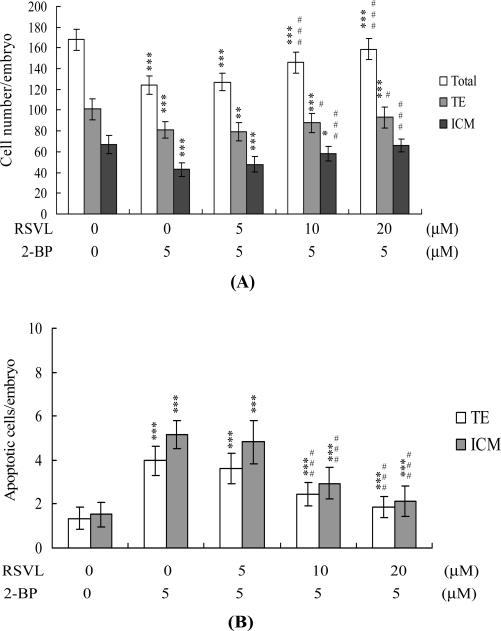
Effect of resveratrol on cell viability in 2-BP-treated blastocysts. Blastocysts were pre-incubated with 5–20 μM RSVL for 1 h, followed by treatment without or with 5 μM 2-BP for a further 24 h. (**A**) The total number of cells per blastocyst and cell numbers in the inner cell mass (ICM) and trophectoderm (TE) were counted; (**B**) The percentages of Annexin V-positive/PI-negative cells in the blastocysts of each group were examined. Data are based on at least 250 blastocyst samples from each group. * *P* < 0.05 and *** *P* < 0.001 *versus* the control group; ^#^ *P* < 0.05 and ^###^ *P* < 0.001 *vs.* the 5 μM 2-BP-treated group.

We initially examined anti-cytotoxicity of resveratrol in mouse embryos on 2-BP exposure. Blastocysts were treated with 2.5–10 μM 2-BP for 24 h, and the level of apoptosis analyzed. 2-BP promoted apoptosis in mouse blastocysts in a dose-dependent manner. Quantitatively, apoptosis was 7.5–11-fold more prevalent in 2-BP-treated blastocysts than in untreated controls (Figure 1A). However, apoptotic cells were not evident in blastocysts treated with 5–20 μM resveratrol (Figure 1A). We further examined the effects of resveratrol on 2-BP-promoted cell death in mouse blastocysts. 2-BP-induced apoptosis was prevented by pretreatment with 10–20 μM resveratrol (Figure 1B). Differential staining followed by cell counting was used to examine cell proliferation within blastocysts to explore the protective effects of resveratrol on cells treated with 2-BP (5 μM) for 24 h. ICM and TE cell numbers were significantly lower in blastocysts treated with 5 μM 2-BP, compared to cell numbers in controls (Figure 2A). Staining revealed significantly higher numbers of Annexin V-positive/PI-negative (apoptotic) cells in the ICM and TE of treated blastocysts, compared to controls (Figure 2B). These results show that 2-BP induces a significant level of apoptosis in the ICM and TE, of mouse blastocysts. Importantly, 2-BP-induced reduction in cell proliferation and apoptosis in blastocysts was effectively prevented by pretreatment with 10–20 μM resveratrol (Figures 2A and 2B). These findings collectively indicate that 2-BP causes embryonic apoptosis and reduced cell proliferation *in vitro*, both of which are effectively prevented by resveratrol.

**Figure 3.**
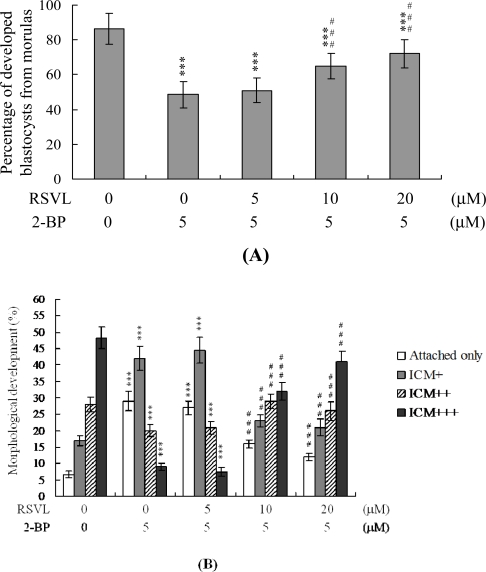
Effect of resveratrol on *in vitro* embryonic development in 2-BP-treated blastocysts. (**A**) Mouse morulae were preincubated with RSVL (5–20 μM) for 1 h, followed by treatment with 5 μM 2-BP for another 24 h. Morulae were cultured at 37 °C, and the percentages of blastocysts counted for 24 h after treatment. Data are based on at least 220 samples in each group; (**B**) Mouse blastocysts were treated with RSVL (5–20 μM) for 1 h or left untreated, followed by 5 μM 2-BP for another 24 h. Blastocysts were observed in culture for 72 h post-treatment. Morphological assessment was used to identify the blastocysts attached with fibronectin-coated dishes only, and classified as ICM (+), ICM (++), and ICM (+++), as described in Materials and Methods. The total blastocyst numbers are 250 for each group. *** *P* < 0.001 *vs.* control group; ^###^ *P* < 0.001 *vs.* 5 μM 2-BP-treated group.

We further analyzed the effects of resveratrol and 2-BP on embryonic development *in vitro*. Approximately 86% of cultured morulae developed into blastocysts (Figure 3A). A similar proportion of morular development was observed in the resveratrol-treated group (data not shown). Development of blastocysts from morulae was reduced by 48.5% in the 5 μM 2-BP-treated group, but this impairment was significantly inhibited by pretreatment with 10–20 μM resveratrol (Figure 3A). We further analyzed the protective effect of resveratrol on outgrowth of embryos treated with 5 μM 2-BP *in vitro*. In the control group, most blastocysts attached to and expanded on fibronectin-coated dishes, whereas fewer blastocysts displayed such behavior after treatment with 5 μM 2-BP (*i.e.*, most blastocysts either only attached, or only developed to the ICM+ stage) (Figure 3B). Furthermore, pretreatment with resveratrol prevented 2-BP-induced inhibition of attachment and outgrowth (Figure 3B). Additionally, we observed a significantly lower extent of outgrowth from 5 μM 2-BP-treated blastocysts that reached the ICM+++ stage (thus with a compact structured ICM) than from control blastocysts; this inhibition was prevented by pretreatment with resveratrol (Figure 3B).

**Figure 4.**
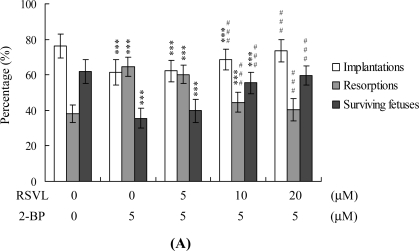

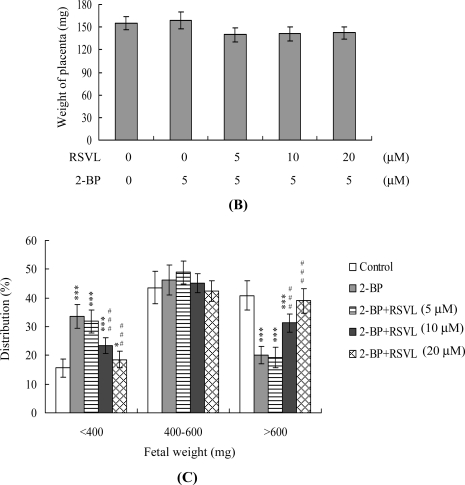
Effect of resveratrol on *in vivo* implantation, resorption, fetal survival and fetal weight in 2-BP-treated blastocysts. (**A**) Mouse morulae were preincubated with RSVL (5–20 μM) for 1 h, followed by treatment with 5 μM 2-BP for another 24 h. Implantations, resorptions and surviving fetuses were analyzed, as described in Materials and Methods. The percentage of implantations represents the number of implantations per number of transferred embryos ×100. The percentage of resorptions or surviving fetuses denotes the number of resorptions or surviving fetuses per number of implantations ×100; (**B**) The placental weights of 40 recipient mice were measured; (**C**) The weight distribution of surviving fetuses on day 18 post-coitus. Surviving fetuses were obtained by embryo transfer of control, RSVL and 2-BP-pretreated blastocysts, as described in the Materials and Methods (320 total blastocysts across 40 recipients). * *P* < 0.05 and *** *P* < 0.001 *versus* the control group; ^###^ *P* < 0.001 *vs.* 5 μM 2-BP-treated group.

To examine the effects of resveratrol and 2-BP on blastocyst development *in vivo*, we transferred mouse blastocysts pretreated with 5 μM 2-BP, or control blastocysts, and examined uterine contents at 13 days post-transfer (thus: on day 18 post-coitus). The implantation ratio in the 5 μM 2-BP-pretreated group was lower than that in untreated controls (Figure 4A). Moreover, the proportion of implanted embryos that failed to develop normally, resulting in embryo resorption in uterus, was significantly higher in the 2-BP-treated group (127 of 197 implanted embryos; 64.5%) than in controls (93 of 244 implanted embryos; 38.1%) (Figure 4A). However, pretreatment with 10–20 μM resveratrol significantly suppressed 2-BP-induced disruption of embryonic development in the embryo transfer assay model (Figure 4A). No difference in placental weight was observed between the 5 μM 2-BP-treated and control groups (Figure 4B), although average fetal weight was lower in the 2-BP-treated group than in controls (479 ± 55 mg *vs.* 619 ± 71 mg, respectively). Consistent with our recent findings, 35–40% of fetuses weighed over 600 mg, and the average weight of total surviving fetuses was ∼600 ± 12 mg, in the untreated control group at day 18 of pregnancy [29–33]. Fetal weight is an important indicator of developmental status. Accordingly, we used average fetal weight as a key marker of the effect of 2-BP on blastocyst development. In our experiments, only 20% of fetuses in the 5 μM 2-BP-treated group weighed over 600 mg, whereas 40.8% of control fetuses exceeded this threshold (Figure 4C). Importantly, 39% of fetuses weighed over 600 mg in the group treated with both 20 μM resveratrol and 5 μM 2-BP (Figure 4C).

**Figure 5.**
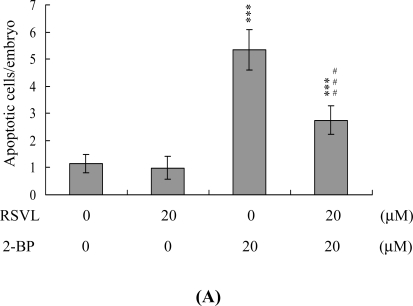

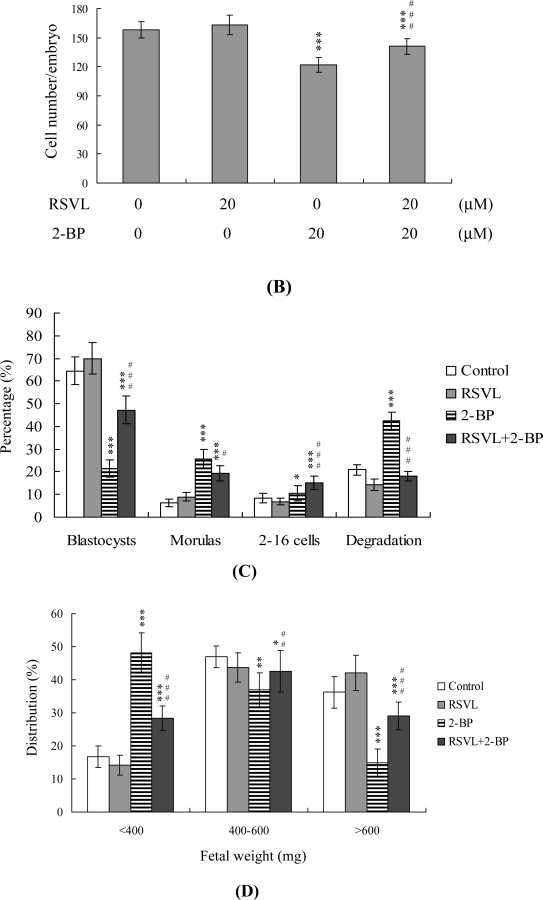
Effects of dietary resveratrol and 2-BP consumption on apoptosis and blastocyst development in an animal model. For the duration of the experiment, randomly selected female mice were fed a standard diet and drinking water supplemented with or without RSVL (20 μM) or/and 2-BP (20 μM). After 24 h, female mice were mated overnight with a single fertile male of the same strain and drinking water continuously supplemented with 2-BP (20 μM) for 4 days. Blastocysts were obtained by flushing the uterine horn on day 4 after mating. (**A**) Apoptosis of mouse blastocysts was examined by TUNEL staining followed by light microscopy, and the mean number of apoptotic (TUNEL-positive) cells per blastocyst calculated; (**B**) The total numbers of cells per blastocyst were counted; (**C**) Developmental stages were compared in embryos obtained from the mouse uterine horns on day 4. Data are presented as the percentage of total embryo obtained; (**D**) The weight distribution of surviving fetuses on day 18 post-coitus was measured. * *P* < 0.05, ** *P* < 0.01 and *** *P* < 0.001 *vs.* the untreated control group; ^#^ *P* < 0.05, ^##^ *P* < 0.01 and ^###^ *P* < 0.001 *vs.* the 2-BP-treated group.

Next, we examined the effects of resveratrol on 2-BP-induced disruption of blastocyst development in an animal model. Female mice were fed a standard diet and the drinking water contained either or both of resveratrol (20 μM) and 2-BP (20 μM). 2-BP consumption induced significant apoptosis and decreased the level of cell proliferation in mouse blastocysts (Figure 5A, B). In addition, 2-BP inhibited embryonic development to the blastocyst stage, resulting in frequent termination at the 2–16 cell or morula stage, or embryo degradation (Figure 5C). Dietary resveratrol effectively reduced these effects of 2-BP (Figure 5A–C). Fetal weight was lower in the 2-BP-treated group than in controls, but was similar to that of control animals in the group receiving both resveratrol and 2-BP. Compared to the control group, the 2-BP-pretreated group contained fewer fetuses weighing over 600 mg (15%), whereas 29.1% of fetuses weighed over 600 mg in the group treated with resveratrol and 2-BP (Figure 5D).

**Figure 6.**
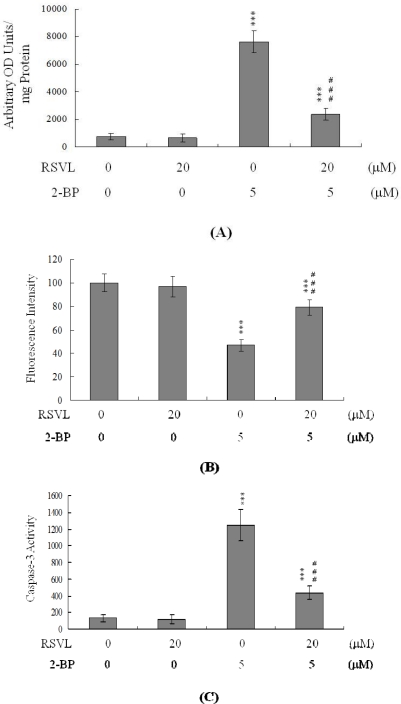
Effects of resveratrol on 2-BP-induced ROS generation and mitochondrion-dependent apoptotic processes in mouse blastocysts. Blastocysts were pre-incubated with 20 μM RSVL for 1 h, followed by treatment with or without 5 μM 2-BP for a further 24 h. (**A**) ROS generation was assayed using 20 μM DCF-DA, and expressed as absorbance/mg of protein. The embryo number per treatment group was 200; (**B**) Analysis of mitochondrial membrane potential changes using 40 nM DiOC_6_ (3); (**C**) Embryos extracts (4 μg) were analyzed for caspase-3 activity using Z-DEVD-AFC as the substrate. Values are presented as means ± SEM of five determinations. Data are based on at least 250 blastocyst samples from each group. *** *P* < 0.001 *vs.* the untreated control group; ^###^ *P* < 0.001 *vs.* the 2-BP-treated group.

In light of recent findings, we conclude that ROS effectively induce apoptosis [33–35]. Moreover, a recent study pointed out that 2-BP impaired cellular antioxidation capacity and increased lipid peroxidation, which is inhibited by antioxidants such as melatonin [8,36]. In the present study, we used a fluorescent dye, DCF-DA, to measure ROS content in resveratrol- and 2-BP-treated mouse blastocyst cells. As shown in Figure 6A, 5 μM 2-BP induced an increase in fluorescence intensity in mouse blastocysts, compared with untreated control cells, and pretreatment with 20 μM resveratrol effectively blocked ROS generation (Figure 6A). To explore the effects of resveratrol on mitochondrial membrane potential (MMP) changes in 2-BP-treated mouse blastocyst cells, we showed that pre-treatment with 20 μM resveratrol blocked suppression of DiOC_6_ (3) uptake into the mitochondria of mouse blastocyst cells, indicative of resveratrol-mediated protection against significant MMP loss in blastocysts (Figure 6B). In addition, 5 μM 2-BP significantly stimulated caspase-3 activation, an important indicator of apoptosis, and this was inhibited by pre-treatment with resveratrol (Figure 6C). Based on these data, we suggest that 2-BP triggers ROS generation, in turn activating mitochondrion-dependent apoptotic processes, in mouse blastocyst cells. Resveratrol abolishes 2-BP-induced apoptosis and injury to blastocysts, because the material scavenges ROS.

Embryonic development is a complex process during which chemical injury can lead to developmental problems or embryonic malformation. 2-BP has been shown to induce apoptosis, to impair oocyte maturation and fertilization, to affect blastocyst development from the morula, and to promote early-stage death of mouse blastocysts [14,37]. Thus, it is important to block the teratogenic effects of 2-BP in female workers exposed to the material. Here, we show, for the first time, that resveratrol significantly inhibits 2-BP-induced cell apoptosis and reduction of cell proliferation (Figures 1 and 2). Moreover, changes in the levels of cell cycle regulators, including PCNA and Ki67, were determined using real-time PCR in 2-BP-treated blastocysts. Expression of both proliferation markers was downregulated after treatment with 5 μM 2-BP, which was effectively prevented by resveratrol (data not shown). Thus, our results indicate that 2-BP significantly inhibits the proliferation of mouse blastocysts, and this effect is blocked by resveratrol. In our preliminary HPLC study, the mice’s drinking water containing 20 μM 2-BP for 4 days had serum 2-BP levels of about 5.63 μM [37]. Our current experiments clearly confirm the injurious effects of 5–10 μM 2-BP. Specifically, 2-BP in this concentration range induces apoptosis, causes a decrease in cell proliferation, and disrupts embryonic development, both *in vitro* and *in vivo* (Figures 1–4). In addition, drinking water with 20 μM 2-BP had adverse effects on both pre- and post-implantation embryonic development in an animal model (Figure 5). Clearly, 2-BP was hazardous to embryonic development at doses reflecting the physiological concentrations that may be attained through female workers exposed to this material. Importantly, pretreatment of 2-BP-treated embryos with resveratrol *in vitro*, or consumption of drinking water containing resveratrol *in vivo,* effectively suppressed 2-BP-induced effects on embryonic development (Figures 3–5).

TE arises from the trophoblast at the blastocyst stage, and develops into a sphere of epithelial cells surrounding the ICM and blastocoel. These cells contribute to the placenta, and are required for development of the mammalian conceptus [38], signifying that a reduction in TE cell lineage may reduce implantation and embryonic viability [39,40]. Interestingly, in our experiments, 2-BP induced apoptosis in the ICM and TE, and had deleterious effects on the rate of implantation *in vivo* (Figures 2 and 4). However, this implantation and development injury was significantly prevented by resveratrol (Figure 4). Previous studies have reported that TE contributes to implantation and placenta formation. Moreover, ∼ 30% or greater reduction in cell number in the ICM is associated with high risk of fetal loss or developmental injury, even in cases where implantation rate and TE cell numbers are normal [41]. In addition, the ICM cell number is essential for proper implantation, and reduction in the ICM lineage may decrease embryonic viability [39,40]. Apoptosis is responsible for eliminating unwanted cells during normal embryonic development, but does not normally occur at the blastocyst stage [42,43]. Excessive apoptosis before or during the blastocyst stage may result in the deletion of important cell lineages, impacting embryonic development and potentially leading to miscarriage or embryonic malformation [44]. In view of our observation that 2-BP reduces cell number and increases apoptosis in the ICM and TE of mouse blastocysts, we further investigated the possibility that the compound causes mortality and/or developmental delay in postimplantation mouse embryos *in vitro* and *in vivo* (Figures 2 and 4) and examined the potential of resveratrol in preventing this injury.

A recent study found that 2-BP induces DNA damage, impairs functional antioxidant cellular defenses, and enhances the lipid peroxidation process in primary cultures of rat Leydig cells [8]. In addition, studies also found that pretreatment with melatonin protected against 2-BP-induced sperm injuries such as morphology, biochemical and histopathological features and apoptosis through its ROS scavenging and anti-apoptotic effects [36]. Oxidative stress is now recognized as a stimulator of cell responses such as apoptosis. Not only can direct exposure of cells to oxidative stress induce apoptosis, but antioxidants also protect cells against apoptosis induced by various stimuli that do not exert direct oxidant effects [45,46]. Moreover, addition of various anti-oxidants to culture media to prevent oxidative stress-induced damage of embryos can improve embryo development [47,48]. Using embryo culture media supplemented with anti-oxidant precursors such as β-mercaptoethanol, cysteamine and vitamin E is beneficial for embryonic inner cell mass (ICM) development [49,50]. In this report, we showed that 2-BP-induced hazardous effects on embryonic development could be blocked by resveratrol in mouse blastocysts (Figures 3–5). Importantly, our study further found that 5 μM 2-BP directly induced ROS generation and through mitochondrion-dependent apoptotic pathway to trigger cell death in mouse blastocysts (Figure 6). These findings indicate that 2-BP can directly evoke ROS generation and apoptosis. Thus, identification of ROS scavengers in dietary foods will be important for prevention of 2-BP-triggered inhibition of embryonic development. Previously, pretreatment with melatonin effectively prevented 2-BP-induced reproductive toxicity in rats through its antioxidant properties [36]. These results imply that 2-BP cause embryonic development injury through ROS and its hazardous effects can be prevented by antioxidants.

The inhibition by resveratrol of the apoptotic biochemical changes triggered by various stimuli have been attributed to the antioxidant properties of the material [28,51–53]. Resveratrol exerts a powerful antioxidant effect against multiple forms of ROS (e.g., O_2_^−^ and H_2_O_2_) produced in macrophages stimulated with lipopolysaccharides or phorbol esters, which induce O_2_^−^ synthesis via the NADPH oxidase pathway [54]. In addition, the specific effects of resveratrol on ethanol-induced ROS generation, intracellular ATP levels, and apoptosis or necrosis in K562 cells, may be dose-dependent [52]. Furthermore, resveratrol prevents ethanol-induced impairment of embryonic development [28]. These previous findings, in conjunction with our current results, clearly indicate that resveratrol attenuates ethanol- or 2-BP-induced ROS generation and apoptosis in mouse embryos, supporting the hypothesis that the compound suppresses cell death via inhibition of ROS production. Importantly, pretreatment with resveratrol could not fully prevent impairment by 2-BP but only reduced the hazardous effects of 2-BP on embryo development, possibly due to the following reasons: (1) the treatment dosage and time-period of resveratrol were not optimal and require further examination; (2) the hazardous effects of 2-BP on embryo development may be exerted through not only ROS but also other injury mechanisms, which cannot be prevented by resveratrol. The underlying regulatory mechanisms thus need further investigation.

## 3. Experimental Section

### 3.1. Chemicals

Pregnant mare serum gonadotropin (PMSG), bovine serum albumin (BSA), sodium pyruvate, resveratrol and 2-Bromopropane were purchased from Sigma (St. Louis, MO, USA). Human chorionic gonadotropin (hCG) was obtained from Serono (NV Organon Oss, the Netherlands). The TUNEL *in situ* cell death detection kit was purchased from Roche (Mannheim, Germany), and CMRL-1066 medium from Gibco Life Technologies (Grand Island, NY, USA).

### 3.2. Collection of Mouse Morulae and Blastocysts

ICR mice were acquired from the National Laboratory Animal Center (Taiwan, ROC). Our research was also approved by the Animal Research Ethics Board of Chung Yuan Christian University (Taiwan, ROC). All animals received humane care, as outlined in the Guidelines for Care and Use of Experimental Animals (Canadian Council on Animal Care, Ottawa, 1984). Mice were maintained on breeder chow (Harlan Teklad chow), with food and water available *ad libitum*. Animals were housed in standard 28 cm × 16 cm × 11 cm (height) polypropylene cages with wire-grid tops, and maintained under a 12 h day/12 h night regimen. Nulliparous females (6–8 weeks old) were superovulated via injection of 5 IU PMSG, followed by 5 IU hCG injection 48 h later, and mated overnight with a single fertile male of the same strain. The day of vaginal plug identification was defined as “day 0” of gestation. Plug-positive females were separated for experimentation. Morulae were obtained by flushing uterine tubes on the afternoon of gestation day 3, and blastocysts acquired by flushing the uterine horn on day 4. In both cases, the flushing solution consisted of CMRL-1066 culture medium containing 1 mM glutamine and 1 mM sodium pyruvate. The glucose concentration in this medium was 5 mM. Expanded blastocysts from different females were pooled and randomly selected for experiments.

### 3.3. Resveratrol and 2-BP Treatments and TUNEL Assay

Blastocysts were incubated in medium containing various concentrations of resveratrol (5, 10 or 20 μM) for 1 h, followed by treatment with 2-BP (2.5, 5 or 10 μM) for a further 24 h. For apoptosis detection, embryos were washed in 2-BP-free medium, fixed, permeabilized and subjected to TUNEL labeling using an *in situ* cell death detection kit (Roche Molecular Biochemicals, Mannheim, Germany), according to the manufacturer’s protocol. Photographic images were obtained under bright-field illumination using a fluorescence microscope.

### 3.4. Measurement of Cell Proliferation

Blastocysts were incubated in medium containing various concentrations of resveratrol (5, 10 or 20 μM) for 1 h, followed by treatment with 2-BP (2.5, 5 or 10 μM) for a further 24 h. Next, blastocysts were washed with 2-BP-free medium, and dual differential staining used to facilitate counting of cell numbers in the inner cell mass (ICM) and trophectoderm (TE) [39]. Blastocysts were incubated in 0.4% pronase in M_2_-BSA (M_2_ medium containing 0.1% bovine serum albumin) for removal of the zona pellucida. Denuded blastocysts were exposed to 1 mM trinitrobenzenesulphonic acid (TNBS) in BSA-free M_2_ medium containing 0.1% polyvinylpyrrolidone (PVP) at 4 °C for 30 min, and washed with M_2_ medium [55]. Further treatment of blastocysts with 30 μg/mL anti-dinitrophenol-BSA complex antibody in M_2_-BSA at 37 °C for 30 min was followed by incubation in M_2_ supplemented with 10% whole guinea pig serum as a source of complement, along with 20 μg/mL bisbenzimide and 10 μg/mL propidium iodide (PI), at 37 °C for 30 min. Immunolysed blastocysts were gently transferred to slides and protected from light before observation. Under UV light excitation, ICM cells (that take up bisbenzimide but exclude PI) appeared blue, whereas TE cells (that take up both fluorochromes) appeared orange-red. Since multinucleated cells are not common in preimplantation embryos [56], the number of nuclei represented an accurate measurement of cell number.

### 3.5. Annexin V Staining

Blastocysts were incubated in medium containing various concentrations of resveratrol (5, 10 or 20 μM) for 1 h, and treated with 2-BP (2.5, 5 or 10 μM) for a further 24 h. For apoptotic staining, embryos were washed in 2-BP-free medium and stained using an Annexin V-FLUOS staining kit (Roche), according to the manufacturer's instructions. Briefly, blastocysts were incubated in M_2_-BSA for removal of the zona pellucida, washed with PBS plus 0.3% BSA, and incubated for 60 min with a mixture of 100 μL binding buffer, 1 μL fluorescein isothiocyanate (FITC)-conjugated Annexin V and 1 μL PI. After incubation, embryos were washed and photographed under fluorescent illumination. Annexin V+/PI- stained cells were considered apoptotic, and Annexin V+/PI+ cells necrotic.

### 3.6. Morphological Analysis of Embryonic Development

Blastocysts were cultured according to a modification of the previously reported method [57]. Briefly, embryos were cultured in 4-well multidishes at 37 °C. For group culture, four embryos were cultured per well. The basic medium consisted of CMRL-1066 supplemented with 1 mM glutamine and 1 mM sodium pyruvate plus 50 IU/mL penicillin and 50 mg/mL streptomycin (hereafter called culture medium). For treatments, the embryos were cultured with the presence or absence of resveratrol (5, 10 or 20 μM) for 1 h, and treated with various concentrations of 2-BP (2.5, 5 or 10 μM). After 24 h, treated blastocysts were individually transferred to fibronectin-coated culture wells and grown for 72 h in CMRL-1066 medium supplemented with 20% fetal bovine serum (CMRL-FBS) [58]. Thereafter, the embryos were cultured for 3 days in culture medium supplemented with 20% fetal calf serum, and for 4 days in culture medium supplemented with 20% heated-inactivated human placental cord serum, for a total culture time of 8 days from the onset of treatment. Embryos were inspected daily under a phase-contrast dissecting microscope, and developmental stages were classified according to established methods [58,59]. Under these culture conditions, each hatched blastocyst attached to the fibronectin and grew to form a cluster of ICM cells over the trophoblastic layer via in a process called TE outgrowth. After a total incubation period of 96 h, morphological scores for outgrowth were estimated. Growing embryos were classified as either ‘attached’ or ‘outgrowth’, with the latter defined by the presence of a cluster of ICM cells over the trophoblastic layer. As described previously [28,60], ICM clusters were scored according to shape, ranging from compact and rounded ICM (+++) to a few scattered cells (+) over the trophoblastic layer.

### 3.7. Blastocyst Development Following Embryo Transfer

To examine the ability of expanded blastocysts to implant and develop *in vivo*, generated embryos were transferred to recipient mice. ICR females (white skin color) were mated with vasectomized males (C57BL/6J; black skin color; National Laboratory Animal Center, Taiwan, ROC) to produce pseudopregnant dams as recipients for embryo transfer. To ensure that all fetuses in pseudopregnant mice came from embryo transfer (white color) and not fertilization by C57BL/6J (black color), we examined the skin color of fetuses at day 18 post-coitus. For assessment of the effects of resveratrol and 2-BP on postimplantation growth *in vivo*, blastocysts were exposed to resveratrol (5, 10 or 20 μM) for 1 h, and treated with 2-BP (2.5, 5 10 μM) for 24 h, following which 8 embryos were transferred in parallel to the paired uterine horns of day 4 pseudopregnant mice. Surrogate mice were killed on day 18 post-coitus, and the frequency of implantation calculated as the number of implantation sites per number of embryos transferred. The incidence rates of resorbed and surviving fetuses were calculated as the number of resorbed or surviving fetuses, respectively, per number of implantations. Weights of surviving fetuses and placenta were measured immediately after dissection.

### 3.8. ROS Assay

ROS were measured in arbitrary units using the DCF-DA fluorescent dyes. Briefly, Embryos were incubated in 20 μM DCF-DA for 1 h at 37 °C, and relative ROS units were determined with a fluorescence ELISA reader (excitation at 485 nm, emission at 530 nm). An aliquot of each cell suspension was lysed, and the protein concentrations were determined using a BCA protein assay kit (Pierce, Rockford, IL). The results are expressed as arbitrary absorbance units/mg protein.

### 3.9. Detection of the Mitochondrial Membrane Potential (MMP) and Caspase-3 Activity

Embryos were exposed to the fluorescent dyes DiOC_6_ (3) for 15 min, and fluorescence was measured with a plate spectrofluorometer (excitation at 485 nm and emission at 535 nm). Caspase-3 activity was measured using the fluorogenic substrate Z-DEVD-AFC. Activation of caspase-3 was inhibited by Acetyl Asp-Glu-Val-Asp aldehyde (Ac-DEVD-cho) (Calbiochem, La Jolla, CA).

### 3.10. Statistical Analyses

Data were analyzed using one-way ANOVA, and t-tests presented as mean values ± SEM, with significance at *P* < 0.05.

## 4. Conclusions

In conclusion, resveratrol, a natural food compound, prevents 2-BP-caused embryonic development injury via blockage of ROS generation, confirming the potential of the material for development as a health food supplement to prevent 2-BP-induced teratogenic effects.
